# Synthesis, Characterization, and Ecotoxicology Assessment of Zinc Oxide Nanoparticles by In Vivo Models

**DOI:** 10.3390/nano14030255

**Published:** 2024-01-24

**Authors:** Ileska M. Casiano-Muñiz, Melissa I. Ortiz-Román, Génesis Lorenzana-Vázquez, Félix R. Román-Velázquez

**Affiliations:** Department of Chemistry, University of Puerto Rico, Mayaguez Campus, Mayaguez, PR 00681, USA; melissa.ortiz10@upr.edu (M.I.O.-R.); genesis.lorenzana@upr.edu (G.L.-V.)

**Keywords:** nanoparticles, toxicology, ecotoxicology, hazard, ZnO, risk, nanomaterials

## Abstract

The widespread use of zinc oxide nanoparticles (ZnO NPs) in multiple applications has increased the importance of safety considerations. ZnO NPs were synthesized, characterized, and evaluated for toxicity in *Artemia salina* and zebrafish (*Danio rerio*). NPs were characterized by X-ray diffraction (XRD), Fourier-transform infrared spectroscopy (FT-IR), and ultraviolet-visible (UV-Vis) spectroscopy. The hydrodynamic size and stability of the ZnO NP surface were examined using a Zetasizer. Characterization techniques confirmed the ZnO wurtzite structure with a particle size of 32.2 ± 5.2 nm. Synthesized ZnO NPs were evaluated for acute toxicity in *Artemia salina* using the Probit and Reed and Muench methods to assess for lethal concentration at 50% (LC50). The LC50 was 86.95 ± 0.21 μg/mL in *Artemia salina*. Physical malformations were observed after 96 h at 50 μg/mL of exposure. The total protein and cytochrome P450 contents were determined. Further analysis was performed to assess the bioaccumulation capacity of zebrafish (*Danio rerio*) using ICP-OES. ZnO NP content in adult zebrafish was greater in the gastrointestinal tract than in the other tissues under study. The present analysis of ZnO NPs supports the use of *Artemia salina* and adult zebrafish as relevant models for assessing toxicity and bioaccumulation while considering absorption quantities.

## 1. Introduction

Zinc oxide nanoparticles (ZnO NPs) are widely used in biomedical applications because of their anti-cancer and antibacterial properties. They are also used as food preservatives [1]; however, they are most commonly used in commercial products because of their unique photocatalytic, electronic, optical, dermatological, and sunscreen capacities [2]. ZnO NPs are semiconductor-type particles with a band gap energy of 3.37 eV [3], corresponding to the wavelength of 368 nm, which corresponds to 6% of the sunlight reaching the Earth’s surface [4]. The hexagonal wurtzite structure is the most abundant, owing to its stability under environmental conditions. 

Owing to the widespread use of ZnO NPs, they reach water bodies through runoff and accumulate in sediments. Numerous studies have documented the aggregation and sedimentation of ZnO NPs ranging from 37 to 100 nm [5,6]. The experiments employed a suspended concentration of 100 mg/L to validate the accumulation in sediments. This leads to their classification as environmental contaminants with potential ecotoxicological risks. These NPs pose a threat to aquatic organisms, as indicated by research [7], and could potentially emerge as a new category of hazardous materials for humans through food webs [8]. Several studies have investigated the toxicity of ZnO NPs in aquatic organisms. ZnO NPs have caused morphological alterations and a significant decrease in human cell viability [9]. Bai W. et al. studied zebrafish embryo toxicity, and tests revealed that ZnO NPs killed zebrafish embryos at 50 and 100 mg/L, retarded embryo hatching (1–25 mg/L), reduced the body length of larvae, and caused tail malformation after 96 h post fertilization (hpf) [10]. In addition, the study suggests that a ZnO NP suspension (47.3 ± 12.9 nm) might permeate into zebrafish eggs through the pore canals, but the large ZnO NP aggregates absorbed into the chorion exceed the canal diameter, and penetration becomes impossible [10]. 

Although there is extensive research on the toxicity of NPs, the content and toxicity of ZnO NPs remain unclear. All available experimental data differ in particle size, stability, pH, type of organism, and exposure time, among others. Aquatic environments are affected by industrial, domestic, and anthropogenic activities. There is a clear lack of research on the specific risks to human health and the environment associated with ZnO NPs used in commercial products. Therefore, it is essential to investigate the fate and behavior of these nanoparticles in the marine environment and examine their biological effects on seawater organisms [11] to increase knowledge regarding their toxicity. ZnO nanoparticles exhibit a higher affinity for binding and absorption on cell surfaces due to their increased surface area. This characteristic has the potential to impede the protein transport pathway across cell membranes [12]. Additionally, the presence of ZnO has been observed to inhibit the population growth rate of phytoplankton, suggesting the possibility of trophic web alterations and a cascade effect on the entire ecosystem [13]. 

It is important to predict and understand the fate, bioavailability, and impact of NPs on aquatic microorganisms and their transfer through the trophic food chain [14]. The use of aquatic models is an alternative to elucidate the ecotoxicology and human risks associated with the use of nanomaterials. The importance of this study is the development of acute toxicity methods using aquatic models (zebrafish and *Artemia salina*) to contribute to the development of environmental studies and human risk regarding exposure to ZnO NPs. Insufficient investigations have been conducted on the effects of ZnO NP usage on specific adult zebrafish, as well as the associated risks to human health and the environment. To study the toxicity of ZnO NPs, *Artemia salina* and adult zebrafish were selected for in vivo studies. 

*Artemia salina* is an invertebrate that has been used for several years in ecotoxicology studies [15]. The 48 h old *Artemia salina* is a suitable choice for bioassays involving its larvae. This developmental stage is optimal for evaluating the lethality of contaminants during the initial developmental stages [15]. Despite its significance, there has been limited exploration of the toxicity of ZnO NPs in *Artemia salina* [11]. The 48 h old *Artemia salina* can tolerate fluctuations in salinity, ion composition, nutrient levels, and diverse chemical concentrations [16]. Additionally, it adapts as a non-selective filter [17]. Notably, the nauplii *Artemia salina* can thrive by consuming particles ranging from 1 to 50 μm in size, providing an ideal platform for studying nanomaterials for ecotoxicology. 

Zebrafish is a small tropical freshwater fish that possesses considerable tolerance to a wide variety of husbandry conditions [18]. These organisms have been used as general toxicity models and ecotoxicological test species to determine the effects of chemicals on fish survival, growth, and reproduction because of their small size and ability to absorb compounds. The high fecundity of zebrafish (each female can lay approximately 200 eggs per week) can generate hundreds of embryos for screening, each of which has a very rapid development [19]. Zebrafish models have been used in multiple research areas, principally in toxicology studies [20]. The analysis of adult zebrafish allows for the application of environmental chemistry practices by establishing contamination through direct exposure to different concentrations and exposure times. 

One of the research objectives involved the synthesis of ZnO nanoparticles. While various methods for synthesizing ZnO NPs have been documented by several researchers [21,22,23], our study employs the polyol process to control both shapes and nanoparticle size [24]. The primary aim of this study was to assess the acute toxicity of synthesized ZnO NPs in both *Artemia salina* and adult zebrafish. Additionally, we investigated the absorbed content of suspended ZnO NPs at various concentrations and exposure durations by detecting and quantifying zinc using ICP-OES analysis. By focusing on these aquatic models, specifically *Artemia salina* and zebrafish, we aimed to advance our understanding of the toxic effects of nanomaterials, particularly ZnO NPs. We used *Artemia salina* to assess acute toxicity, and adult zebrafish were used as a model to evaluate the bioaccumulation capacity of various fish tissues after exposure to different concentrations of ZnO NPs. 

The novelty of this work lies in utilizing in vivo models to evaluate total protein content, cytochrome P450, and zinc adsorption in adult zebrafish tissues when exposed to ZnO NPs. Notably, no ecotoxicity research has been conducted on synthesized and characterized nanoparticles within the same research article. Furthermore, there is a dearth of prior studies investigating morphological changes in the digestive tract of *Artemia salina* after exposure to ZnO NPs with a size of less than 40 nm.

## 2. Materials and Methods

### 2.1. ZnO Nanoparticle Synthesis

The ZnO NPs were synthesized using a previously published method [25] by precipitation of a mixture of two aqueous solutions: a 0.4 M solution of zinc acetate dihydrate (Zn (CH_3_COO)·2H_2_O, 98 ± 2% Alfa Aestar, Haverhill, MA, USA), 0.004 M of polyvinylpyrrolidone (PVP; 10,000 M.W.), with 725 µL of deionized water, and 50 mL of diethylene glycol (DEG, (HOCH_2_CH_2_)_2_O, 99% as a solvent, Sigma Aldrich, St. Louis, MO, USA). The working temperature was set at 180 °C and refluxed for 30 min. The obtained solutions were centrifuged at 8000 rpm, washed with ethanol three times, and stirred at ambient temperature until a white precipitate appeared. The white precipitate was transferred to a crystal petri dish and heated at 60 °C on a hot plate for two hours to obtain the ZnO NP powder. The NPs were stored at room temperature prior to the characterization and toxicity experiments. 

### 2.2. Preparation of Aqueous Suspensions

For the determination of the *Artemia salina* LC50, ZnO NP solutions were created by suspending the appropriate powder amounts in brine shrimp artificial seawater. The seawater was prepared by adding 60 mg of sea salt (Instant Ocean^®^ Sea Salt, Blacksburg, VA, USA) per mL of deionized water (18 MΩ·cm, pH 7.0 ± 0.5). The final concentrations of suspended ZnO NPs ranged from 10 to 400 µg/mL (10, 20, 50, 80, 100, 200, and 400 µg/mL). It is noteworthy that *Artemia salina* were exposed to concentrations higher than those typically found in the environment, such as surface water (0.001 to 0.058 µg/L) and sewage treatment plant effluents (0.33 to 1.42 µg/L) [26]. The NP suspension solutions were dispersed using a Bransonic 3510R-MT ultrasonic processor (Branson Ultrasonics Corp., Danbury, CT, USA) (frequency: 40 kHz) for 15 min to achieve the maximum dispersion before use. For the *Artemia salina* cytochrome P450 total content, ZnO NPs were dispersed under the same experimental conditions to obtain final concentrations of 2.5, 10, and 50 µg/mL. For zebrafish exposure and chemical analysis, ZnO NPs were dispersed for 15 min in system water (SW) prepared with a controlled salinity (1000 ± 100 µS/cm) and pH (7.0 ± 5.0) to final concentrations of 2.5, 10, 50, and 100 µg/mL by using the same equipment as previously described. The control groups were prepared without ZnO NPs under the same experimental conditions.

### 2.3. Nanoparticle Characterization

ZnO NPs were characterized at ambient temperature using various techniques. The optical absorption of the ZnO NPs dispersed in deionized water and system water was evaluated using UV-Vis absorption spectrophotometry. These assessments were performed with a Thermo Scientific Evolution 220 UV-Visible spectrophotometer (Thermo Scientific, Waltham, MA, USA) within the spectral range of 300–600 nm. Quartz cuvettes with a path length of 1 cm were used, and the spectral resolution was set to 1 nm.

To determine the hydrodynamic size and zeta potential value of the ZnO NPs (60 μg/mL), a Zetasizer Nano ZS90 system (Malvern Instrument Zetasizer Software 6.12) was used. Disposable polystyrene cuvettes with a sample volume of 1 mL were used for measurements. The measurement conditions included a temperature of 25 °C, a sample equilibration time of 120 s, a dispersant (water) refractive index of 1.330, a viscosity of 0.8872 cP, a dispersant dielectric constant of 78.5, and a laser source wavelength of 532 nm. The Zetasizer system employed dynamic light scattering (DLS) to analyze the Brownian motion of the NPs, and these data were then interpreted using established theories to determine their size. Furthermore, the instrument assesses electrophoretic mobility as the method for determining the zeta potential of the ZnO NPs. 

X-ray diffraction (XRD) was used to identify the composition, size, deformation, and orientation of the samples using a SIEMENS D 500 diffractometer. The XRD pattern was recorded using CuKa radiation (wavelength λ = 1.54 Å) (generated at 40 kV and 40 mA) and compared with the Joint Committee on Power Diffraction Standards (JCPDS).

Fourier-transform infrared spectroscopy (FTIR) was used to study the vibrational behavior of the synthesized metal-oxygen NPs. The analysis was performed using a PerkinElmer Spectrum (PerkinElmer, Hong Kong, China) Two spectrophotometer with an attenuated total reflectance (ATR) device attached. The spectra were recorded in the range of 4000 to 490 cm^−1^. 

### 2.4. Artemia salina Acute Toxicity Test

#### 2.4.1. Lethal Concentration at 50%

*Artemia salina* (nauplius stages) brine shrimp cysts were hydrated for one hour in deionized water. Hatching was performed within 48 h of incubation in brine shrimp water. Proper aeration was provided through an aquarium air pump and light illumination (10 W, 0.44 mW/cm^2^). Hatched brine shrimp larvae were used for toxicity testing. 

Acute toxicity tests were performed according to the protocols of Reed and Muench [27] and Probit [28]. After 48 h of incubation, ten *Artemia salina* were introduced into test tubes containing suspended ZnO NPs at concentrations of 10, 20, 50, 80, 100, 200, and 400 µg/mL. No additional food was provided during the period, and the experiment included six replicates for subsequent analysis. *Artemia salina* were used for toxicity studies of lethal concentrations at 50 percent (LC50). The control group was prepared in the absence of target ZnO NPs under the same experimental conditions. The test tubes were maintained at 25 ± 1 °C for 48 h. After 48 h, the number of dead individuals was counted, and the LC50 was calculated. Validation of the results was guaranteed by a control group, which may show less than 10% mortality. After the experiment, *Artemia salina* were euthanized with 70% ethanol (Sigma Aldrich). 

#### 2.4.2. *Artemia salina* Morphological Changes 

*Artemia salina* (nauplius stages) brine shrimps were exposed to 0, 2.5, 10, and 50 µg/mL of ZnO NPs for 24, 48, 72, and 96 h (10 individuals per sample) and examined for toxicity using a stereoscope (AmScope SM-2T2-LED, Irvine, CA, USA) and were randomly distributed to different cover glass slides. They were observed for development, appearance, and abnormal behavior after acute toxicity.

#### 2.4.3. Measurement of the Total Content of Cytochrome P450 in *Artemia salina*

*Artemia salina* (nauplius stages) brine shrimp cysts were hydrated for one hour in deionized water, followed by hatching within 48 h of incubation in brine shrimp water. Adequate aeration was maintained using an aquarium air pump, and the experiment was conducted under light illumination.

*Artemia salina* brine shrimps were subjected to ZnO NPs at concentrations of 0, 2.5, 10, and 50 µg/mL (with approximately 10,000 individuals per sample) for 24, 48, 72, and 96 h. Subsequently, the samples were examined for toxicity.

*Artemia salina* were euthanized before the cytochrome P450 assay for 15 min with 70% ethanol (Sigma Aldrich). Each group of exposed *Artemia salina* was homogenized with seven volumes of ice-cold homogenization buffer prepared with 0.1 M sodium phosphate (Sigma Aldrich), pH 7.50, 1 mM ethylene diamine tetra acetic acid (EDTA; Sigma Aldrich), 0.1 mM dithiothreitol (Sigma Aldrich), and phenylmethylsulfonyl fluoride (PMSF; Sigma Aldrich) supplemented with 10% (*v*/*v*) glycerol (Sigma Aldrich) using a glass homogenizer at 4 °C to prevent enzyme breakdown. The homogenate was centrifuged at 13,000 rpm for 30 min in a refrigerated centrifuge (SL 16R; Thermo Scientific, Waltham, MA, USA) at 4 °C. The supernatant was centrifuged at 13,000 rpm for 3 h at 4 °C. The microsomal pellet was resuspended in a homogenization buffer supplemented with 20% (*v*/*v*) glycerol. The resulting microsomal suspension was stored at 4 °C the following day. Microsomal preparations were performed as previously described by Dong et al. [18]. 

The total protein content of each sample was determined using the Bradford protein assay [29]. A bovine serum albumin (BSA) calibration curve was prepared by measuring the absorbance at 595 nm using an Implen nanophotometer P330. Standards were prepared using a stock solution of 1000 µg/mL BSA (Sigma Aldrich), and dilutions of 0, 1, 1.5, 5, 10, and 40 µg/mL BSA were prepared using distilled water. Five milliliters of Bradford reagent (Sigma-Aldrich) were added to each standard to a final volume of 6 mL. For the samples, 160 µL of distilled water, 500 µL of Bradford reagent, and 50 µL of microsomes were added to a 2 mL centrifuge tube. The samples were incubated at room temperature for 15 min before analysis, and the absorbance was measured at 595 nm. The total protein content of each sample was determined by extrapolating standard curves. The blue color appeared as an indicator of protein content in the samples. 

The total cytochrome P450 content was determined according to the method described by Omura and Sato [30]. The *Artemia salina* microsomes were mixed in a vortex (Daigger Vortex Genie 2, Daigger Scientific, Inc., Hamilton, NJ, USA) for 15 s, and 50 μL of the microsomes was diluted in 550 μL of resuspension buffer (pH 7.4) prepared with 50 Mm Tris buffer (Fisher bioreagents, Fisher Scientific, Hampton, NH, USA) supplemented with 20% (*v*/*v*) glycerol (Sigma Aldrich), 1 mM EDTA (Sigma Aldrich), and 1 mM dithiothreitol (Sigma Aldrich), and bubbled with CO (count of 10; Sigma Aldrich). Baseline correction was performed between 400 nm and 600 nm. Sodium dithionite (Sigma Aldrich; 2 mg) was added to each sample, and the absorbance was measured from 400 to 600 nm. The difference between the absorbance at 450 nm and 490 nm was used to calculate the cytochrome P450 content. The total cytochrome content was measured at 91 mM^−1^ cm^−1^ and expressed as nmol mg^−1^ of protein. 

### 2.5. Zebrafish Regular Care and Maintenance

Adult zebrafish (*Danio rerio*) aged between one and two years were obtained from Caribe Fisheries, Inc., Lajas, Puerto Rico. Zebrafish were maintained at 25 ± 1 °C in groups of 20 ± 5 fish per tank in the benchtop husbandry unit (Techniplast) at the University of Puerto Rico in Mayagüez, with an artificial light regimen of 12:12 h (dark: light). They were kept in reverse osmosis water prepared with commercial sea salts (Instant Ocean^®^ Sea Salt, USA) with a conductivity of 1000 ± 100 µS, mechanical filtration, and sterilization (ultraviolet disinfection at 40 W). NaHCO_3_ was added to maintain a pH of 7.40 ± 0.5. The fish were fed twice daily with a Zeigler adult zebrafish diet. 

### 2.6. Zebrafish Exposure

Groups of 15 adult zebrafish were exposed to varying concentrations of nanoparticles (0, 2.5, 10, 50, and 100 µg/mL) over a 10-day period in two litter tanks with continuous aeration. Water was changed every two days to minimize treatment variation. The zebrafish were fed twice daily, and pH levels were monitored throughout the exposure. After the 10-day exposure, each group of 15 adult zebrafish was euthanized in ice-cold water (2–4 °C) for 20 min before dissection. The samples were analyzed in triplicate, each consisting of 5 fish. In total, 75 adult zebrafish were used for the experiment. Dissections included the gastrointestinal tract, gills, skin, and eggs, following the method outlined by Gupta and Mullins [31]. Organ samples, ranging from 0.0143 to 0.1138 g, were placed in 15 mL centrifuge tubes and stored at −20 °C.

### 2.7. Sample Preparation and Quantification of ZnO NPs

**Scheme 1:**
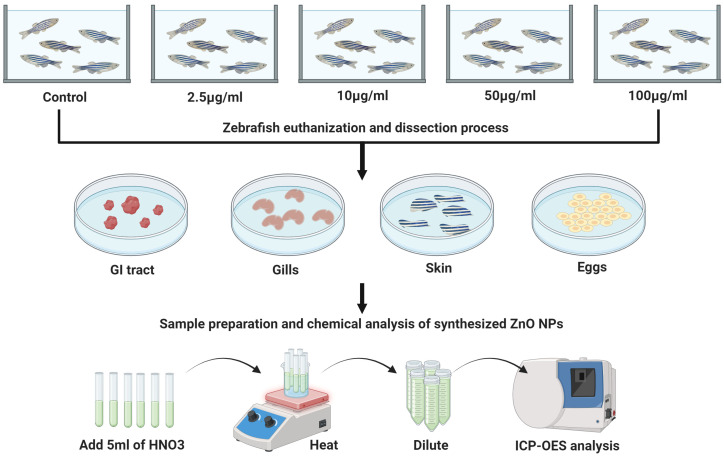
Experimental settings for adult zebrafish. Zebrafish were exposed to ZnO NPs from 2.5 to 100 μg/mL per 10 days. Subsequently, the zebrafish were euthanized and dissected. The gastrointestinal tract, gills, skin, and eggs were removed for further content analysis. The concentration of zinc was determined using ICP-OES analysis. Created with BioRender.com.

To determine the ionic portion of the zinc (Zn^2+^) vs. ZnO NPs in the suspension solution, a combined methodology of digestion and filtration was performed. Zebrafish tissues were mixed with 5 mL of trace metal-grade HNO_3_ (68 to 70%) and heated on a hot plate while controlling the temperature of the mixture at 70–80 °C for 3 h. The solutions were cooled to room temperature for 15 min. The Zn^2+^ concentration was measured at 206.2 nm. A calibration curve was constructed using the Zn^2+^ standard (1000 µg/mL ± 2 µg/mL in nitric acid; Sigma-Aldrich) by preparing dilutions ranging from 0 to 20 µg/mL. The Zn^2+^ sample concentrations were converted using a stoichiometric conversion factor of 1.24 ZnO (Scheme 1). 

### 2.8. Statistical Analysis

Statistical significance between each treatment solution and the control was determined using one-way analysis of variance (ANOVA) for total protein content, cytochrome P450 content, and the quantification of ZnO in adult zebrafish tissue. Least significant differences (LSD) were employed to compare differences between the groups. Significance was established at *p* < 0.05, and the critical F value exceeded the calculated F value. All data are presented as the mean ± standard deviation (SD). Microsoft Excel version 2311 was utilized for statistical analysis.

### 2.9. Ethics Statement

All experiments at the University of Puerto Rico, Mayaguez Campus, that implicated the use of wild-type zebrafish adults were conducted with the approval of the local Institutional Animal Care and Use Committee (IACUC) in agreement with the Office of Laboratory Animal Welfare assurance number D20-01098, following the guidelines of the National Research Council Guide for the Care and Use of Laboratory Animals.

## 3. Results and Discussion

### 3.1. ZnO NPsCharacterization

ZnO NPs have been synthesized for further applications regarding the acute toxicity of *Artemia salina* and adult zebrafish. The suitability of the synthesized ZnO NPs for toxicity studies was established through a rigorous characterization process that ensured consistency in size, hydrodynamic diameter, surface charge, agglomeration of functional groups, and bonding. The stability of ZnO NPs (core size = 32.2 ± 5.2 nm) in both deionized water and system water was evaluated by measuring the absorbance, hydrodynamic diameter, and zeta potential. 

**Figure 1:**
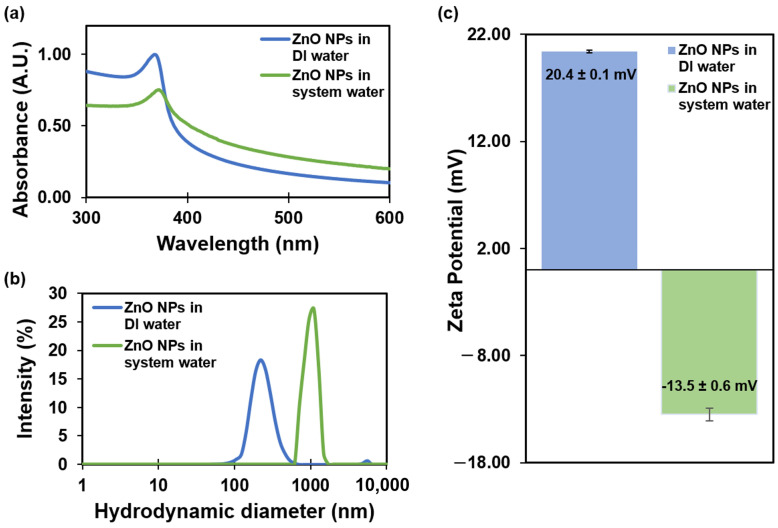
Characterization of ZnO NPs in DI water and system water: (**a**) UV-Vis absorption spectra; (**b**) hydrodynamic diameter; and (**c**) zeta potential results obtained from Zetasizer Nano measurements. Error bars correspond to standard deviations.

The UV-Vis absorption spectra showed a sharp peak at 368 nm for ZnO NPs dispersed in DI water and a peak at 372 nm for ZnO NPs dispersed in system water (Figure 1a). In the case of ZnO NPs in the system water, the UV band exhibited a shift of 4 nm. The noticeable shift exhibited toward a longer wavelength in the UV band of ZnO NPs in the system water is a signal of NP aggregation or increasing size due to the absorption of ions on its surface. Studies have demonstrated that ZnO NPs with an average core particle size of 20–30 nm exhibit UV absorption at a wavelength of approximately 370 nm, indicating a smaller size of the NPs [32]. 

Hydrodynamic diameter measurements obtained using a Zetasizer Nano indicated a size of 237 ± 7 nm for ZnO NPs in DI water, with a polydispersity index (PDI) of 0.237. In contrast, ZnO NPs in the system water exhibited a larger hydrodynamic diameter of 1011 ± 173 nm and a PDI of 0.703 (Figure 1b). The significant increase in hydrodynamic diameter when comparing ZnO NPs in DI water and SW suggests that aggregation is likely induced by the presence of salts in the system water, while the elevated PDI values also point to a high dispersion in particle size. Bhuvaneshwari et al. also observed similar results about the formation of aggregates in seawater [11]. A previous study was conducted to investigate NP agglomeration size in suspensions over time [10]. The results indicate changes in size, ranging from 47.3 ± 12.9 to 142.4 ± 36.8 nm at 1 mg/L and from 101.4 ± 26.4 to 260.8 ± 41.9 nm at 10 mg/L after 24 h. 

Zeta potential measurements indicated a positive zeta potential value of 20.4 ± 0.1 mV for ZnO NPs in DI water (pH 7.6 ± 0.2 and conductivity 0.06 μS/cm). In contrast, ZnO NPs in the system water (pH 7.6 ± 0.2 and conductivity 1000 ± 200 μS/cm) displayed a negative zeta potential value of −13.5 ± 0.6 mV (Figure 1c). The reduction in the potential value was also influenced by the presence of electrolytes and the higher conductivity in the SW. Previous studies have demonstrated similar results, in which 50 nm ZnO NPs in DI water exhibited a hydrodynamic size of 209 ± 7 nm with a lower PDI, whereas ZnO NPs in a water-based salt solution displayed a hydrodynamic size of 970 ± 276 nm with a high PDI and a negative potential charge [33]. Our hydrodynamic results were consistent with previous findings.

**Figure 2:**
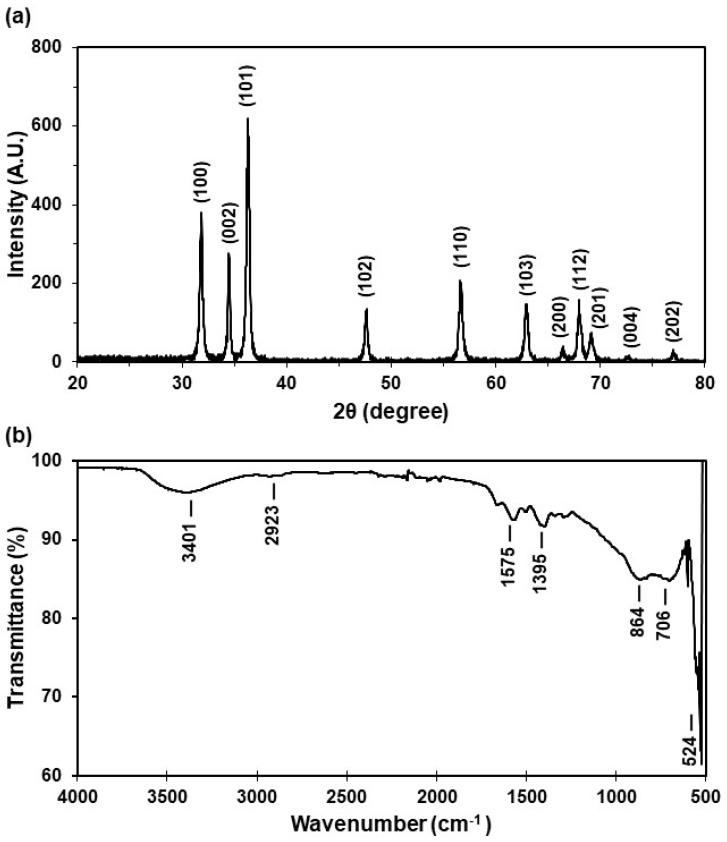
Characterization of the ZnO NP solid powder: (**a**) X-ray diffraction pattern and (**b**) FT-IR spectrum.

The crystalline structure of ZnO NPs was characterized using X-ray diffraction (XRD). The XRD patterns were used to determine the peak intensity, position, and full width at half maximum (FWHM). The diffraction peaks located at 31.8°, 34.5°, 36.3°, 47.6°, 56.6°, 62.9°, 66.4°, 68.0°, 69.4°, 72.9°, and 77.3° can be indexed to the pure hexagonal wurtzite phase structure of ZnO, represented as (100), (002), (101), (102), (110), (103), (200), (112), (201), (004), and (202), respectively, with lattice constants of a = b = 3.24 Å and c = 5.21 Å [34] (Figure 2a). The average grain size (32.2 ± 5.2 nm) of the samples was estimated from the XRD diffractogram using the Scherrer equation (Equation (1)) by using the diffraction intensity of the (101) peak, where l is the wavelength (CuKa), b is the FWHM of the ZnO (101) line, and q is the diffraction angle.

(1) $$d\;=\;\frac{K\lambda }{b\;COS\theta }$$

The X-ray diffraction (XRD) pattern analysis revealed that the peak intensity, position, and FWHM align with the pure hexagonal wurtzite phase structure of ZnO. Significantly, the average NP size measures 32.2 ± 5.2 nm, a crucial parameter for this study. Particles of this size are highly valuable, particularly in applications such as commercially available sunscreens and cosmetics, where semiconducting metal oxide nanoparticles below 50 nm effectively block ultraviolet radiation [12]. Moreover, their size can affect toxicity in aquatic organisms [27].

To identify the specific functional groups and bonds formed during ZnO NP synthesis, Fourier transform infrared (FT-IR) spectroscopy was employed. The resulting spectrum displays absorption peaks at 3401 cm^−1^, 2923 cm^−1^, 1575 cm^−1^, 1395 cm^−1^, 864 cm^−1^, 706 cm^−1^, and 524 cm^−1^. Notably, the band between 470 and 560 cm^−1^ (524 cm^−1^) corresponds to ZnO stretching vibrations (Figure 2b). The peaks at 1395 cm^−1^ and 1575 cm^−1^ indicate C-H stretching, while the peak at 2923 cm^−1^ corresponds to C-O stretching. The FT-IR analysis confirmed the presence of C-O and C-H bond groups, with OH stretching observed around 3500 cm^−1^, attributed to ZnO functionalization. In summary, the FT-IR spectrum robustly confirms the formation of ZnO NPs, elucidating the characteristic functional groups and suggesting potential peaks arising from ZnO functionalization.

### 3.2. Artemia salina Acute Toxicity

#### 3.2.1. Lethal Concentration

**Figure 3:**
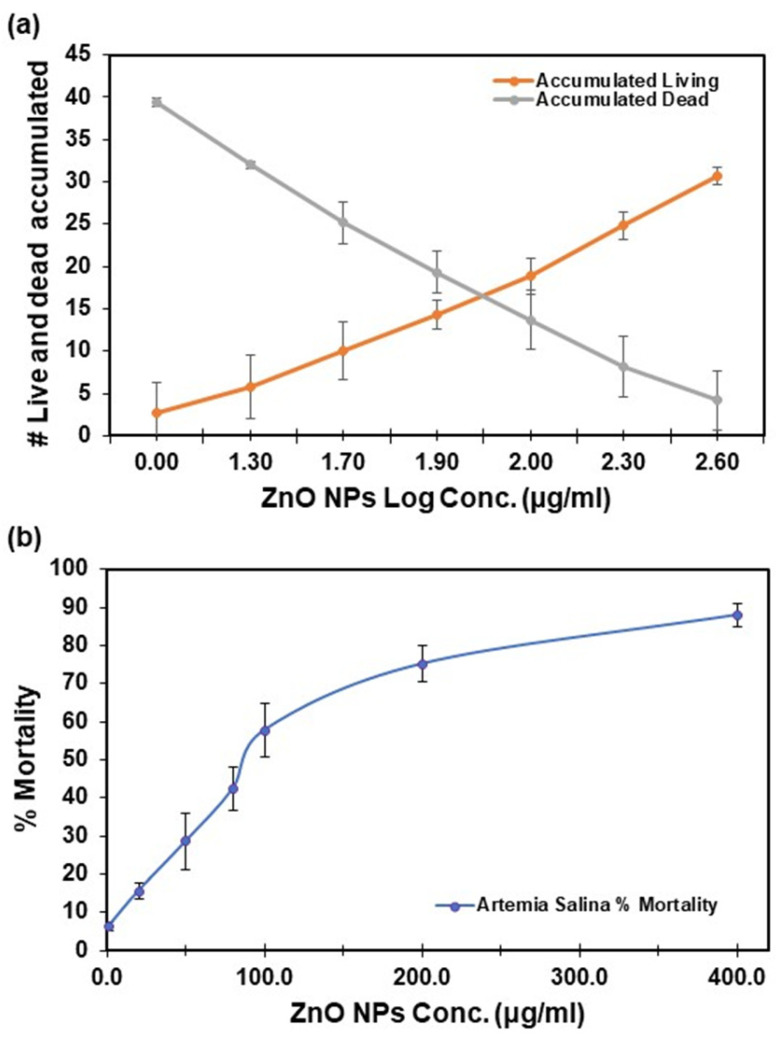
*Artemia salina* acute toxicity for lethal concentrations was calculated using the Reed and Muench method: (**a**) number of live *Artemia salina brine shrimp* and dead accumulated vs. log dose; and (**b**) percentage of mortality vs. ZnO NPs concentration. Error bars represent the mean ± standard deviation of six replicates.

The acute toxicity of ZnO NPs in *Artemia salina* was evaluated through two methods: Reed and Muench, and Probit (refer to Table 1). The toxicity of *Artemia salina* demonstrated a proportional increase with the concentration of ZnO NPs, as depicted in Figure 3a,b. At the highest concentration of ZnO NPs (400 µg/mL), the observed mortality reached 88 ± 3% (Figure 3b). In contrast, at a concentration of 1 µg/mL, the mortality was as low as 6 ± 1%, highlighting a clear dose dependency for ZnO. Lethal concentrations (at 10%, 20%, 30%, 50%, and 80%) were assessed at 48 h, revealing significant differences. It is important to evaluate LC in the range of 10–80% because introducing a xenobiotic leading to a mortality rate exceeding 20% within a population is considered a substantial loss, and this prolonged decline can have cascading effects on interconnected species, resulting in a 20% reduction in the overall food chain. The examination of these values in the range of 10–80% has been documented using other pollutants [35,36], and it is crucial for assessing the impact of xenobiotics on an ecosystem population.

Confidence in the test results was ensured by control samples, which exhibited mortality rates below 10%. Reed and Muench results were averaged from graph estimations and exponential equations.

Several studies have explored the potential impacts of ZnO on aquatic models, employing accumulation, elimination, and uptake studies to evaluate the risk of ZnO NPs [37]. *Artemia salina* is a crustacean involved in the primary food chain transfer in the marine ecosystem [11]. This organism was selected as an experimental model organism due to its resistance to toxic chemicals and its suitability for nanomaterial toxicological testing [38,39]. 

The LC50 results provide valuable insights into the impact of varying concentrations of ZnO on *Artemia salina*. The reported LC50 in our study was 86.95 ± 0.21 µg/mL, while Bhuvaneshwari et al. [11] found an LC50 of 71.63 µg/mL under similar experimental conditions. The number of deceased organisms increased with increasing concentrations of ZnO NPs in the suspension, suggesting dose-dependent toxicity. Danabas et al. [40] and Mehmet et al. [14] also reported LC50 values between 89 and 100 µg/mL, emphasizing the size-dependent toxicity of ZnO NPs. Smaller ZnO NPs (10–30 nm) induced higher mortalities than larger ones (200 nm), corroborating the findings that nanoparticle toxicity may vary even with a similar size distribution [14]. Another study reported an LC50 of 247.13 µg/mL for ZnO NPs with a size distribution of 10 to 30 nm [41]. 

*Artemia salina* are relatively resistant to chemicals, including metal oxides, and its ability to tolerate environmental concentrations suggests that concentrations exceeding the LC50 in *Artemia salina* may induce significant detrimental effects in less tolerant species, such as zebrafish. Zebrafish embryos, for instance, exhibit an LC50 value between 7.1 and 11.9 µg/mL [42]. Therefore, we selected 50 µg/mL of ZnO NPs as the maximum concentration for further analysis of adult zebrafish ZnO NP uptake.

#### 3.2.2. Morphological Changes

**Figure 4:**
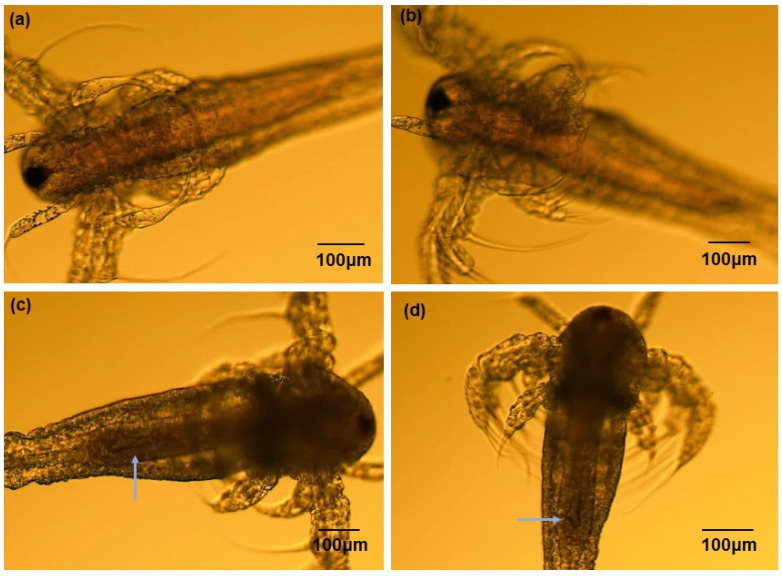
*Artemia salina* acute toxicity morphological changes after exposure to ZnO NPs at different concentration levels for 96 h: (**a**) control, (**b**) 2.5 µg/mL, (**c**) 10.0 µg/mL, and (**d**) 50.0 µg/mL. The arrows indicate physical malformations, including intestinal enlargement.

After exposing *Artemia salina* to ZnO NPs at concentrations of 2.5, 10, and 50 µg/mL for 24, 48, 72, and 96 h, morphological observations were conducted to interpret any changes. Figure 4 illustrates distinctions between the control and treatment groups, particularly in the digestive tract. The control group appeared empty and clear (Figure 4a), whereas the ZnO NP-exposed groups exhibited noticeable symptoms at all tested concentrations. With increasing ZnO NP concentrations, significant differences were observed in the digestive tract, particularly in the 50 µg/mL treatment group (Figure 4d). Microscopic examination revealed physical malformations, including intestinal enlargement. No missing antennae or extremities were observed, and the naupliar eyes showed no notable changes at any treatment concentration. These findings suggest a potential for ZnO NP accumulation in *Artemia salina*, consistent with observations by Ates et al. [14], indicating an inability to eliminate ingested particles. The accumulation of nanoparticles may disrupt body homeostasis and alter diets, potentially accelerating mortality in organisms. 

The results also suggest that *Artemia salina* have the capacity to tolerate changes without discrimination against ZnO NPs. The effect of dissolved Zn^2+^ on the toxicity and accumulation of Zn in *Artemia salina* has also been suggested [11]. Further studies are necessary to visualize specific body areas that may accumulate and identify the target organs affected by ZnO NP exposure.

#### 3.2.3. Total Content of Cytochrome P450

**Figure 5:**
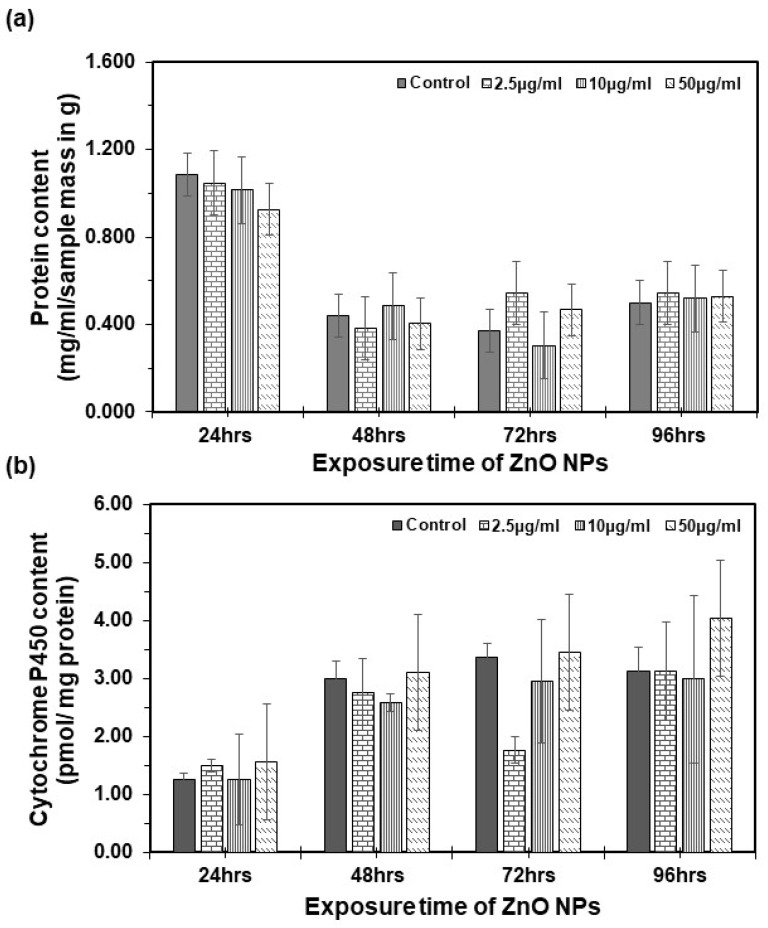
Cytochrome P450 content in *Artemia salina*: (**a**) total protein content per sample determined by the Bradford method. (**b**) Total cytochrome P450 content as determined by *Omura and Sato* [30]. Each bar represents the mean of three replicates, with error bars at the top. Error bars represent the standard deviation (SD) of the mean.

*Artemia salina* were subjected to varying concentrations of suspended ZnO NPs, and the total protein content was assessed using the Bradford protein assay (see Figure 5a). One-way ANOVA analysis conducted at 24, 48, 72, and 96 h revealed no statistically significant difference in total protein content between the control and treatment groups (*p* > 0.05). Similarly, no statistically significant changes were observed in the total cytochrome P450 content following each exposure (refer to Figure 5b). Comparable research in mice indicated minimal toxicity in the expression of CYP450 levels upon exposure to ZnO NPs [43]. Specific isoenzymes of cytochrome P450 were investigated, showing varied activities. Cytochrome P3A1 exhibited lower activity, cytochrome P1A2 did not increase, cytochrome P2C11 was suppressed, and cytochrome P1A1 was induced in the intestine in the presence of ZnO NPs at different concentration levels [44]. This aligns with our findings, indicating no significant differences in cytochrome P450 total content, possibly due to variations in cytochrome enzyme activities.

However, when performing ANOVA for both total protein content and cytochrome P450 total content analysis, a statistically significant difference emerged between the groups across exposure times. Notably, as time increased from 24 to 96 h, there was a decrease in the total protein content and an increase in the total cytochrome P450 content. These observed biochemical changes, coupled with an increase in the mortality rate, may suggest potential disruptions at the biochemical level. Similar changes are observed in the control group, indicating that these differences may be attributed to the normal development of the organism rather than exposure to ZnO NPs.

This study provides novel insights, as no prior research has explored the total cytochrome P450 in *Artemia salina* regarding the acute toxicity of ZnO NPs. While the concentrations under study exhibited no significant toxic effects on cytochrome P450 total content, the observed changes in protein content and potential biochemical disruptions warrant further investigation into the mechanisms underlying ZnO NP toxicity. Future studies may reveal the specific molecular interactions that lead to biochemical alterations.

### 3.3. Quantification of ZnO in Adult Zebrafish Tissue

Zinc content in adult zebrafish tissues was quantified after 10 days of ZnO NP exposure. The detection and quantification of Zn were conducted using ICP-OES after adult zebrafish were exposed to previously synthesized and characterized ZnO NPs. After exposure to 0.0, 2.5, 10.0, 50, and 100 µg/mL, the Zn^2+^ concentration was quantified in different zebrafish tissues: gastrointestinal tract (GI tract), gills, skin, and eggs. Six spikes were performed to determine the accuracy of the chemical analysis method, with recovery percentages ranging from 90.3 to 114.5%. The limits of detection and quantification were 0.4 µg/mL and 1.4 µg/mL, respectively. No Zn^2+^ was detected in the control group. Zn^2+^ was only detected in the gastrointestinal tract (GI tract), gills, and skin. Zinc was not detected in the eggs (Table 2). 

**Figure 6:**
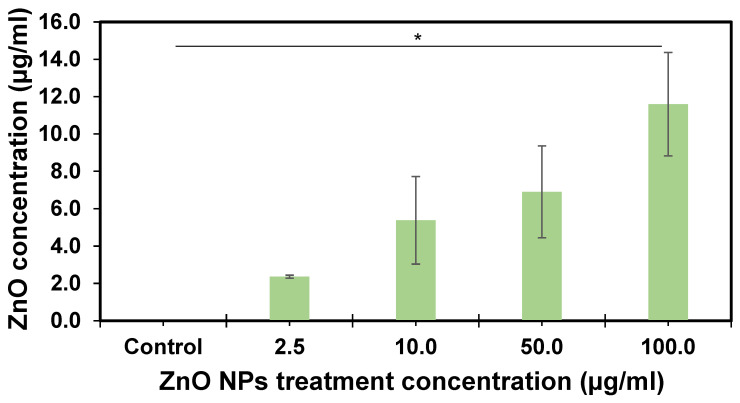
ZnO concentration in the adult zebrafish gastrointestinal tract after ten days of exposure. Each bar represents the mean of three replicates, with error bars at the top. Error bars represent the standard error of the mean (SEM). The asterisk indicates statistical differences (*p* = 0.02) performed by LSD to compare differences between the groups.

Concentration-dependent ZnO NPs were observed in the zebrafish GI tract (Figure 6), as the concentration increased after 10 days of exposure. The maximum zinc content was observed in the GI tract at 11.59 ± 4.7 µg/mL ZnO and 4.65 ± 2.16 in the gills when a dose of 100.0 µg/mL of ZnO nanoparticle exposure treatment was used. These results represent 11.6% in the GI tract and 4.7% in the gills at a total dose of 100 µg/mL (Table S1), representing 16.3% of the total dose. ZnO content in zebrafish GI tract samples was statistically significant (*p* = 0.02) at all concentration levels. The overall tissue analysis results were significantly different (*p* = 0.0003). 

The measured content of ZnO NPs in various zebrafish tissues remains undocumented, revealing a crucial gap in our understanding of their distribution. Concentrations were notably higher in the GI tract than in other tissues, aligning with similar research that identified the gut as a major site for zinc absorption [45]. Previous studies, including one on *Bombyx mori*, demonstrated ZnO NPs crossing the gut barrier, leading to toxic responses such as ROS generation, morphological alterations, and apoptotic cell death [45]. These findings suggest that ingestion is the primary route of chemical exposure. The zebrafish GI tract emerges as a valuable indicator for monitoring emerging contaminants in environmental matrices. Consistent with research on mice, ZnO NPs were found to accumulate in the digestive tract, with higher concentrations in the liver and kidney [46]. Other studies reported intestinal injury in mice due to ZnO ingestion, affecting permeability and allowing the entry of zinc and other substances into the liver [47]. The observed variations in zinc accumulation in tissues indicate that the principal target of ZnO NPs in adult zebrafish is the GI tract, potentially leading to changes in substance absorption and other detrimental effects [48]. 

This study highlights the importance of considering the safety of ZnO NPs and emphasizes the need for comprehensive research in organisms to extrapolate their potential effects in humans. These results support the idea that zinc absorption by organisms is primarily due to dissolved ionic zinc. Recent studies have confirmed the ability of ZnO NPs to release Zn^2+^ into the medium [9], and UV irradiation has been shown to accelerate their dissolution in seawater [49]. The toxicity of ZnO NPs in water is related to the dissolution and release of Zn^2+^ [50]. Additionally, the generation of reactive oxygen species (ROS) emerges as a crucial factor in the toxicity of ZnO NPs. A recent study demonstrated an increase in catalase (CAT) activity in *Artemia salina*, indicating the potential disruption of homeostasis in aquatic organisms owing to nanoparticle interaction [11]. These findings collectively highlight the multifaceted aspects of ZnO NP toxicity and underscore the need for continued research to fully understand their environmental and human health implications. The discovery of our study emphasizes the necessity for continued research on ZnO NP toxicity to comprehend their potential environmental and human health implications.

## 4. Conclusions

The present study investigated the toxic effects of synthesized ZnO NPs (32.2 ± 5.2 nm) in aquatic models, focusing on acute toxicity and potential bioaccumulation effects in *Artemia salina* and adult zebrafish tissues. The study identified significant lethal concentrations and observed physical malformations in the digestive tract of *Artemia salina*. Additionally, ZnO NPs have been detected in fish tissues following exposure to acute toxicity. This study underscores the suitability of *Artemia salina* and zebrafish as alternative models for assessing toxicity and bioaccumulation, offering a quantification method for ZnO NP content using ICP-OES. These findings support the use of these aquatic organisms as valuable tools in toxicity studies.

While providing valuable insights, this study suggests the need for further research on ZnO NPs to enhance our understanding of nanomaterial uptake and its effects on organisms. Future investigations could explore specific mechanisms, potential long-term impacts, and the extrapolation of findings to human health, thereby contributing to a more comprehensive knowledge base in this evolving field.

## Figures and Tables

**Table 1:** *Acute toxicity of Artemia salina*: lethal concentrations at different mortality percentages calculated using different methods.

	Lethal Concentration Values (µg/mL)				
LC Methods	LC10	LC20	LC30	LC50	LC80
Reed and Muench	12.85 ± 0.65	31.48 ± 11.72	53.60 ± 11.82	86.80 ± 8.25	267.44 ± 65.17
Probit	14.34 ± 2.33	26.04 ± 4.14	42.96 ± 6.73	87.10 ± 15.15	273.67 ± 59.48
Average	13.60 ± 1.06	28.76 ± 3.84	48.28 ± 7.53	86.95 ± 0.21	270.56 ± 4.41

The lethal concentration values are presented as the mean ± standard deviation of six replicates.

**Table 2:** Quantified concentrations of ZnO NPs in adult zebrafish tissues (µg/mL).

	Adult Zebrafish Dissected Tissues			
Treatment Concentrations (μg/mL)	GI tract	Gills	Skin	Eggs
Control	ND	ND	ND	ND
2.5	2.37 ± 0.13	ND	ND	ND
10.0	5.38 ± 4.06	ND	ND	ND
50.0	6.90 ± 4.26	D	D	ND
100.0	11.59 ± 4.79	4.65 ± 2.16	ND	ND

ND corresponds to the ZnO concentration that was not detected. D corresponds to the ZnO concentration in samples with values between the LOD and LOQ. All reported values are expressed as the mean ± standard deviation. The analysis was performed in triplicate.

## Data Availability

The datasets generated during the study are available from the corresponding author upon request.

## Supplementary Materials

The following supporting information can be downloaded at: https://www.mdpi.com/article/10.3390/nano14030255/s1, Table S1: ZnO uptake in the gastrointestinal tract.
