# Supplementary material for: Synthesis, Characterization, and Ecotoxicology Assessment of Zinc Oxide Nanoparticles by In Vivo Models
Source: Nanomaterials (Basel). 2024 Jan 24;14(3):255. doi: 10.3390/nano14030255 (PMC10857287; doi:10.3390/nano14030255)
Supplement: Supplementary file 1 [file nanomaterials-14-00255-s001.zip › nanomaterials-2814049-supplementary.pdf]

Supplementary Material

**Table S1** ZnO content at gastrointestinal tract

| Treatment concentrations (μg/ml) | ZnO content | Content percent (%) |
|---|---|---|
| Control | 0 | ND |
| 2.5 | 2.37 ± 0.13 | 94.4 |
| 10.0 | 5.38 ± 4.06 | 53.8 |
| 50.0 | 6.90 ± 4.26 | 13.8 |
| 100.0 | 11.59 ± 2.16 | 11.6 |

ND corresponds to a ZnO concentration non detected. All the reported values are expressed as the mean ± SD. Analysis was performed in triplicate.
